# Medical Journals Are an Extension of the Marketing Arm of Pharmaceutical Companies

**DOI:** 10.1371/journal.pmed.0020138

**Published:** 2005-05-17

**Authors:** Richard Smith

## Abstract

Medical journals have become dependent on the pharmaceutical industry for their survival, which can have a corrupting influence on their content, argues Smith, the former editor of the *BMJ.*

“Journals have devolved into information laundering operations for the pharmaceutical industry”, wrote Richard Horton, editor of the *Lancet*, in March 2004 [1]. In the same year, Marcia Angell, former editor of the *New England Journal of Medicine*, lambasted the industry for becoming “primarily a marketing machine” and co-opting “every institution that might stand in its way” [2]. Medical journals were conspicuously absent from her list of co-opted institutions, but she and Horton are not the only editors who have become increasingly queasy about the power and influence of the industry. Jerry Kassirer, another former editor of the *New England Journal of Medicine*, argues that the industry has deflected the moral compasses of many physicians [3], and the editors of *PLoS Medicine* have declared that they will not become “part of the cycle of dependency…between journals and the pharmaceutical industry” [4]. Something is clearly up.

## The Problem: Less to Do with Advertising, More to Do with Sponsored Trials

The most conspicuous example of medical journals' dependence on the pharmaceutical industry is the substantial income from advertising, but this is, I suggest, the least corrupting form of dependence. The advertisements may often be misleading [5,6] and the profits worth millions, but the advertisements are there for all to see and criticise. Doctors may not be as uninfluenced by the advertisements as they would like to believe, but in every sphere, the public is used to discounting the claims of advertisers.

The much bigger problem lies with the original studies, particularly the clinical trials, published by journals. Far from discounting these, readers see randomised controlled trials as one of the highest forms of evidence. A large trial published in a major journal has the journal's stamp of approval (unlike the advertising), will be distributed around the world, and may well receive global media coverage, particularly if promoted simultaneously by press releases from both the journal and the expensive public-relations firm hired by the pharmaceutical company that sponsored the trial. For a drug company, a favourable trial is worth thousands of pages of advertising, which is why a company will sometimes spend upwards of a million dollars on reprints of the trial for worldwide distribution. The doctors receiving the reprints may not read them, but they will be impressed by the name of the journal from which they come. The quality of the journal will bless the quality of the drug.[Fig pmed-0020138-g001]

**Figure pmed-0020138-g001:**
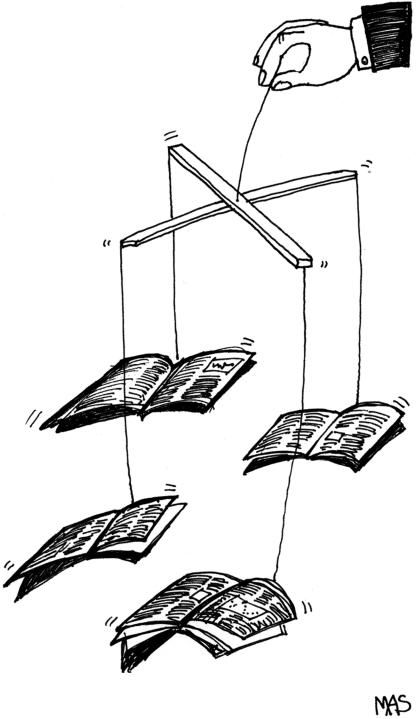
(Illustration: Margaret Shear, Public Library of Science)

Fortunately from the point of view of the companies funding these trials—but unfortunately for the credibility of the journals who publish them—these trials rarely produce results that are unfavourable to the companies' products [7,8]. Paula Rochon and others examined in 1994 all the trials funded by manufacturers of nonsteroidal anti-inflammatory drugs for arthritis that they could find [7]. They found 56 trials, and not one of the published trials presented results that were unfavourable to the company that sponsored the trial. Every trial showed the company's drug to be as good as or better than the comparison treatment.

By 2003 it was possible to do a systematic review of 30 studies comparing the outcomes of studies funded by the pharmaceutical industry with those of studies funded from other sources [8]. Some 16 of the studies looked at clinical trials or meta-analyses, and 13 had outcomes favourable to the sponsoring companies. Overall, studies funded by a company were four times more likely to have results favourable to the company than studies funded from other sources. In the case of the five studies that looked at economic evaluations, the results were favourable to the sponsoring company in every case.

The evidence is strong that companies are getting the results they want, and this is especially worrisome because between two-thirds and three-quarters of the trials published in the major journals—*Annals of Internal Medicine*, *JAMA*, *Lancet*, and *New England Journal of Medicine*—are funded by the industry [9]. For the *BMJ*, it's only one-third—partly, perhaps, because the journal has less influence than the others in North America, which is responsible for half of all the revenue of drug companies, and partly because the journal publishes more cluster-randomised trials (which are usually not drug trials) [9].

## Why Do Pharmaceutical Companies Get the Results They Want?

Why are pharmaceutical companies getting the results they want? Why are the peer-review systems of journals not noticing what seem to be biased results? The systematic review of 2003 looked at the technical quality of the studies funded by the industry and found that it was as good—and often better—than that of studies funded by others [8]. This is not surprising as the companies have huge resources and are very familiar with conducting trials to the highest standards.

The companies seem to get the results they want not by fiddling the results, which would be far too crude and possibly detectable by peer review, but rather by asking the “right” questions—and there are many ways to do this [10]. Some of the methods for achieving favourable results are listed in the Sidebar, but there are many ways to hugely increase the chance of producing favourable results, and there are many hired guns who will think up new ways and stay one jump ahead of peer reviewers.

Then, various publishing strategies are available to ensure maximum exposure of positive results. Companies have resorted to trying to suppress negative studies [11,12], but this is a crude strategy—and one that should rarely be necessary if the company is asking the “right” questions. A much better strategy is to publish positive results more than once, often in supplements to journals, which are highly profitable to the publishers and shown to be of dubious quality [13,14]. Companies will usually conduct multicentre trials, and there is huge scope for publishing different results from different centres at different times in different journals. It's also possible to combine the results from different centres in multiple combinations.

These strategies have been exposed in the cases of risperidone [15] and odansetron [16], but it's a huge amount of work to discover how many trials are truly independent and how many are simply the same results being published more than once. And usually it's impossible to tell from the published studies: it's necessary to go back to the authors and get data on individual patients.

## Peer Review Doesn't Solve the Problem

Journal editors are becoming increasingly aware of how they are being manipulated and are fighting back [17,18], but I must confess that it took me almost a quarter of a century editing for the *BMJ* to wake up to what was happening. Editors work by considering the studies submitted to them. They ask the authors to send them any related studies, but editors have no other mechanism to know what other unpublished studies exist. It's hard even to know about related studies that are published, and it may be impossible to tell that studies are describing results from some of the same patients. Editors may thus be peer reviewing one piece of a gigantic and clever marketing jigsaw—and the piece they have is likely to be of high technical quality. It will probably pass peer review, a process that research has anyway shown to be an ineffective lottery prone to bias and abuse [19].

Furthermore, the editors are likely to favour randomised trials. Many journals publish few such trials and would like to publish more: they are, as I've said, a superior form of evidence. The trials are also likely to be clinically interesting. Other reasons for publishing are less worthy. Publishers know that pharmaceutical companies will often purchase thousands of dollars' worth of reprints, and the profit margin on reprints is likely to be 70%. Editors, too, know that publishing such studies is highly profitable, and editors are increasingly responsible for the budgets of their journals and for producing a profit for the owners. Many owners—including academic societies—depend on profits from their journals. An editor may thus face a frighteningly stark conflict of interest: publish a trial that will bring US$100 000 of profit or meet the end-of-year budget by firing an editor.

## Journals Should Critique Trials, Not Publish Them

How might we prevent journals from being an extension of the marketing arm of pharmaceutical companies in publishing trials that favour their products? Editors can review protocols, insist on trials being registered, demand that the role of sponsors be made transparent, and decline to publish trials unless researchers control the decision to publish [17,18]. I doubt, however, that these steps will make much difference. Something more fundamental is needed.

Firstly, we need more public funding of trials, particularly of large head-to-head trials of all the treatments available for treating a condition. Secondly, journals should perhaps stop publishing trials. Instead, the protocols and results should be made available on regulated Web sites. Only such a radical step, I think, will stop journals from being beholden to companies. Instead of publishing trials, journals could concentrate on critically describing them.

**Examples of Methods for Pharmaceutical Companies to Get the Results They Want from Clinical Trials**

- Conduct a trial of your drug against a treatment known to be inferior.
- Trial your drugs against too low a dose of a competitor drug.
- Conduct a trial of your drug against too high a dose of a competitor drug (making your drug seem less toxic).
- Conduct trials that are too small to show differences from competitor drugs.
- Use multiple endpoints in the trial and select for publication those that give favourable results.
- Do multicentre trials and select for publication results from centres that are favourable.
- Conduct subgroup analyses and select for publication those that are favourable.
- Present results that are most likely to impress—for example, reduction in relative rather than absolute risk.
